# Synaptic vesicle generation from activity‐dependent bulk endosomes requires a dephosphorylation‐dependent dynamin–syndapin interaction

**DOI:** 10.1111/jnc.14862

**Published:** 2019-10-17

**Authors:** Giselle Cheung, Michael A. Cousin

**Affiliations:** ^1^ Centre for Discovery Brain Sciences University of Edinburgh Edinburgh UK; ^2^ Muir Maxwell Epilepsy Centre University of Edinburgh Edinburgh UK; ^3^ Simons Initiative for the Developing Brain University of Edinburgh Edinburgh UK; ^4^ Present address: Institute of Science and Technology Austria Am Campus 1 3400 Klosterneuburg Austria

**Keywords:** calcium, dynamin, endocytosis, endosome, pre‐synapse, vesicle

## Abstract

Activity‐dependent bulk endocytosis generates synaptic vesicles (SVs) during intense neuronal activity via a two‐step process. First, bulk endosomes are formed direct from the plasma membrane from which SVs are then generated. SV generation from bulk endosomes requires the efflux of previously accumulated calcium and activation of the protein phosphatase calcineurin. However, it is still unknown how calcineurin mediates SV generation. We addressed this question using a series of acute interventions that decoupled the generation of SVs from bulk endosomes in rat primary neuronal culture. This was achieved by either disruption of protein–protein interactions via delivery of competitive peptides, or inhibition of enzyme activity by known inhibitors. SV generation was monitored using either a morphological horseradish peroxidase assay or an optical assay that monitors the replenishment of the reserve SV pool. We found that SV generation was inhibited by, (i) peptides that disrupt calcineurin interactions, (ii) an inhibitor of dynamin I GTPase activity and (iii) peptides that disrupt the phosphorylation‐dependent dynamin I–syndapin I interaction. Peptides that disrupted syndapin I interactions with eps15 homology domain‐containing proteins had no effect. This revealed that (i) calcineurin must be localized at bulk endosomes to mediate its effect, (ii) dynamin I GTPase activity is essential for SV fission and (iii) the calcineurin‐dependent interaction between dynamin I and syndapin I is essential for SV generation. We therefore propose that a calcineurin‐dependent dephosphorylation cascade that requires both dynamin I GTPase and syndapin I lipid‐deforming activity is essential for SV generation from bulk endosomes.

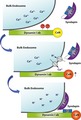

## Graphical abstract

Activity‐dependent bulk endocytosis is the dominant pathway via which synaptic vesicles (SVs) are generated during intense neuronal activity. Calcineurin is essential for SV generation from bulk endosomes and we aimed to determine its mechanism. We found that SV generation was inhibited by: disruption of calcineurin interactions, inhibition of dynamin‐I activity and disruption of dynamin‐I–syndapin‐I interactions. This revealed that 1) calcineurin localization at bulk endosomes, 2) dynamin‐I GTPase activity and 3) calcineurin‐dependent dynamin‐I–syndapin‐I interactions are all essential. We propose that a calcineurin‐dependent dephosphorylation cascade requiring dynamin‐I GTPase and syndapin‐I lipid‐deforming activity is essential for SV generation from bulk endosomes.

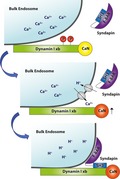

**Abbreviations used**
ADBE: activity‐dependent bulk endocytosis
anova: analysis of variance
BAR: Bin‐amphiphysin‐Rvs
CME: clathrin‐mediated endocytosis
EH: Eps15 homology
EHD: Eps15 homology domain
HRP: horse radish peroxidase
RRP: readily releasable pool
SEM: standard error of the mean
SH3: src homology 3
SV: synaptic vesicle

Normal brain function is reliant on the efficient release of neurotransmitter in response to action potential stimulation. For neurotransmitter release to be sustained, a pool of release‐ready synaptic vesicles (SVs) must be maintained within pre‐synaptic nerve terminals. These pools can be separated into the readily releasable pool (RRP, which are mobilized immediately; Rosenmund and Stevens 1996) and the reserve SV pool (which is only mobilized during intense neuronal activity; Richards *et al.* 2003). These SV pools are maintained by a series of different endocytosis modes that are triggered by specific patterns of neuronal activity. During very sparse periods of activity, ultrafast endocytosis is triggered (Watanabe *et al.* 2013; Watanabe *et al.* 2014), however, this endocytosis mode quickly saturates during action potential trains (Soykan *et al.* 2017). Clathrin‐mediated endocytosis (CME) is also triggered during mild periods of neuronal activity (Granseth *et al.* 2006), however, this mode also saturates during high bursts of high‐frequency firing (Clayton *et al.* 2008). During these specific periods, activity‐dependent bulk endocytosis (ADBE) is the dominant endocytosis mode (Clayton *et al.* 2008), forming bulk endosomes direct from the plasma membrane from which SVs are then generated (Kokotos and Cousin 2015). Both ultrafast and CME refill the RRP (Granseth and Lagnado 2008; Watanabe *et al.* 2014), whereas ADBE exclusively replenishes the reserve SV pool (Cheung *et al.* 2010).

ADBE is triggered by activity‐dependent calcium influx, which stimulates the calcium‐dependent protein phosphatase calcineurin (Evans and Cousin 2007; Clayton *et al.* 2009; Wu *et al.* 2009; Sun *et al.* 2010; Wu *et al.* 2014a; Morton *et al.* 2015). Calcineurin dephosphorylates the large GTPase dynamin I, allowing an interaction with syndapin I (Anggono *et al.* 2006). The recruitment of calcineurin to dynamin I is mediated via a specific splice variant (dynamin Ixb) which has a C‐terminal PRITIS motif (Xue *et al.* 2011). Depletion of endogenous syndapin I using shRNA also arrests ADBE (Clayton *et al.* 2009), however, how syndapin I mediates this process is still undetermined. Syndapin I is a modular protein that possesses a lipid‐deforming F‐Bin‐amphiphysin‐Rvs (BAR) domain, two central NPF repeats that interact with Eps15 homology (EH) domains and a C‐terminal src homology 3 (SH3) domain that is the site of interaction with dynamin I (Qualmann *et al.* 1999; Braun *et al.* 2005; Wang *et al.* 2009).

ADBE generates bulk endosomes, from which SVs are produced to replenish the reserve SV pool (Cheung *et al.* 2010). The molecular mechanism of SV generation is still relatively unknown; however, clathrin and related adaptor proteins perform a key role (Cheung and Cousin 2012; Kononenko *et al.* 2014). Calcineurin activity is also essential for SV generation from bulk endosomes (Cheung and Cousin 2013). The efflux of accumulated extracellular calcium from bulk endosomes activates calcineurin during their acidification (Cheung and Cousin 2013). However, several questions remain unaddressed. For example how is calcineurin localized to bulk endosomes to encounter this localized calcium efflux? In addition, what is the substrate of calcineurin and what is the consequence of its dephosphorylation?

A recent proteomic study from our group catalogued the molecules present on bulk endosomes (Kokotos *et al.* 2018). Intriguingly, calcineurin, dynamin I and syndapin I were all present, suggesting they may perform parallel roles in SV generation at the bulk endosome. In this study, we demonstrate that both calcineurin–dynamin I and dynamin I–syndapin I interactions are essential for SV generation at bulk endosomes. Dynamin I appears to be the fission mediator, since the dynamin inhibitor dynasore inhibits SV generation and competitive peptides that interfere with the syndapin–EH domain proteins (EHDs) interaction have no effect. We therefore propose that a calcineurin‐dependent dephosphorylation cascade that requires both dynamin GTPase and syndapin I lipid‐deforming activity is essential for SV generation from bulk endosomes.

## Materials and methods

Penicillin/streptomycin (Cat # 15140‐122), phosphate‐buffered salts (21300‐058) and Minimal Essential Medium (Cat # 21090‐022) were purchased from Invitrogen (Paisley, Scotland, UK). Foetal bovine serum (Cat # FB‐1001/500, lot # 013BS715) was from Biosera (Nuaille, France). KCl (Cat # BP366) was from Fluka UK (Gillingham, UK). Peptides were synthesized by Genemed Synthesis (San Antonio, TX, USA). The peptides used in this study were as follows – DynIxb_842–851_ – GVPRITISDP; DynIxb_842–851AAA_ –GVARATASDP; DynI_769–784_AA – PAGRRAPTSAPTPQRR; DynI_769–784_EE – PAGRREPTSEPTPQRR; SydI_356–381_ – EWSDDESGNPFGGNEANGGANPFEDD; SydI_356–381AAA_ – EWSDDESGAAAGGNEANGGAAAAEDD. All peptides were fused to the sequence – RRMKWKK – that permits entry into cells (Lindgren *et al.* 2000; Cousin *et al.* 2003) and reconstituted in distilled water. Glutaraldehyde (Cat # R1020) and osmium tetroxide (Cat # AGR1022) were from Agar Scientific (Essex, UK). All other reagents were from Sigma (Poole, UK).

### Preparation of cerebellar granule neuron cultures

All animal work was performed in accordance with the UK Animal (Scientific Procedures) Act 1986, under Project (PPL – 7008878) and Personal Licence (PIL – I7C942245) authority and was approved by the Animal Welfare and Ethical Review Body at the University of Edinburgh. Specifically, all animals were killed by pentobarbital anaesthetic overdose, with death confirmed via the destruction of the brain. In‐house Sprague–Dawley rat breeding colonies (original source; Charles River, Saffron Walden, UK) were housed in standard open top caging on a 14 h light/ dark cycle (light 07:00–21:00 hours) and were maintained on RM3 chow.

Cerebellar granule neuron cultures were prepared from the pooled cerebella of 7‐day‐old rat pups of both sexes as previously described (Tan *et al.* 2003). Briefly, neurons were plated on poly‐d‐lysine (Cat # P7886) coated glass coverslips at a density of 0.25 × 10^6^ cells/ coverslip. Neurons were cultured in Minimal Essential Medium, plus 10% (v/v) foetal bovine serum, 25 mM KCl, 30 mM glucose (Cat # G8270), 2 mM glutamine (Cat # G7029), 100 U/mL penicillin and 100 µg/mL streptomycin at 37 °C, in a humidified atmosphere of 5% CO_2_: 95% air. Culture medium was supplemented with 10 µM cytosine arabinoside (Cat # C1768) after 24 h *in vitro*. In all cases cultures were used between 8 and 10 days *in vitro*.

### Labelling of endocytic pathways by HRP

**Figure 1:**
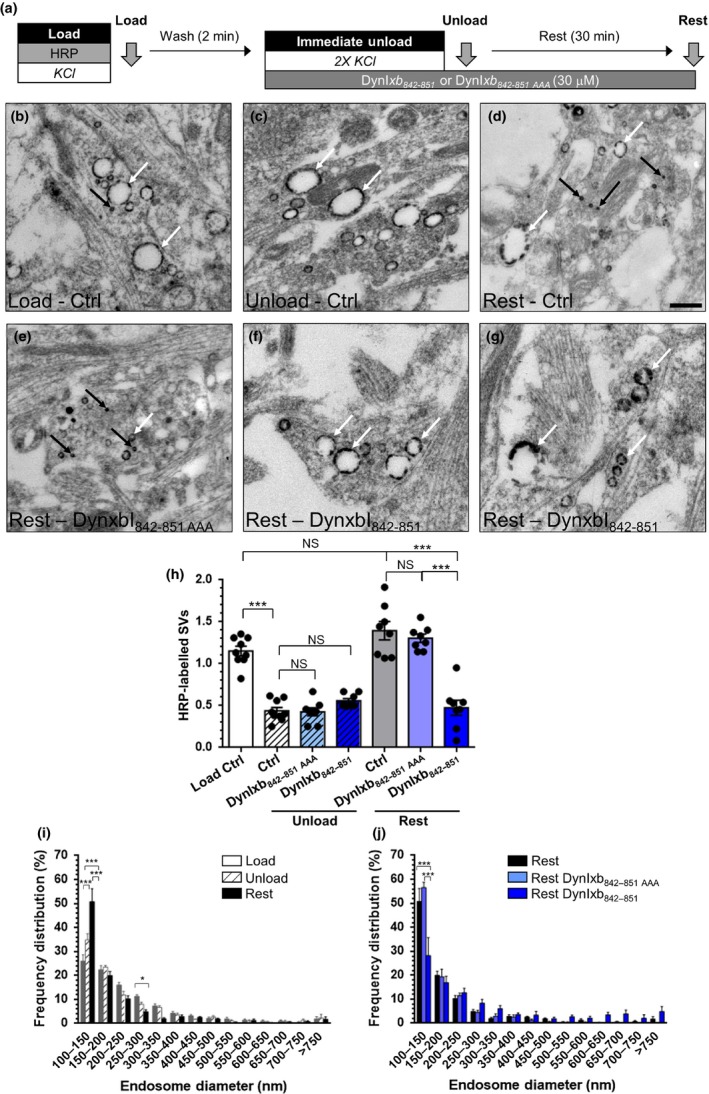
Disruption of calcineurin interactions arrest synaptic vesicle (SV) budding from bulk endosomes. (a) Cultures were loaded with horse radish peroxidase (HRP) (10 mg/mL) for 2 min in the presence of KCl (50 mM) and washed immediately to remove excess HRP. Where indicated cultures were incubated with either DynIxb_842–851_ or DynIxb_842–851AAA_ peptides (both 30 µM) immediately after this wash step. Neurons were then stimulated twice with KCl (50 mM, 30 s each) to release all available SVs (Immediate Unload) and left to rest for 30 min. Cultures were either fixed after HRP loading (Load), the immediate unload (Unload) or the rest period (Rest) as indicated by arrows. (b–g) Representative electron micrographs of the treatments described above are shown (b – Load, c‐ Unload, d – Rest, e – Rest DynIxb_842–851AAA_, f, g – Rest DynIxb_842–851_; scale bar – 500 nm). Black and white arrows indicate HRP‐labelled SVs and endosomes respectively. (h) Bar graph displays the mean number of HRP‐labelled SVs per nerve terminal ± SEM in either Load, Immediate Unload (hatched bars) and Rest (solid bars) conditions in the presence or absence of peptides. Number of experiments: *n* = 8 for all conditions except Rest Ctrl and Unload Ctrl which was *n* = 9 (all from three culture preparations; ***p < 0.001 one‐way anova). (i, j) Frequency distribution of endosome diameter for Load, Unload and Rest in the absence of competitive peptides (i) or Rest with competitive peptides (j). The number of HRP‐labelled endosomes were as follows: (i) Load *n* = 2338; Unload *n* = 1459; Rest *n* = 983; *n* = 7 coverslips from three culture preparations; (j) Rest *n* = 983; Rest DynIxb_842–851AAA_ *n* = 443; Rest DynIxb_842–851_ *n* = 415; *n* = 9 coverslips, from three culture preparations, except Rest Ctrl which was *n* = 8. Two‐way anova for both I and J (*p < 0.05, ***p < 0.001).

Cultures were fixed and processed for electron microscopy as described (Cheung *et al.* 2010). Briefly, cultures were placed in incubation medium [in mM: 170 NaCl, 3.5 KCl, 0.4 KH_2_PO_4_, 20 TES (*N*‐tris[hydroxyl‐methyl]‐methyl‐2‐aminoethane‐sulphonic acid), 5 NaHCO_3_, 5 glucose, 1.2 Na_2_SO_4_, 1.2 MgCl_2_ and 1.3 CaCl_2_; at pH 7.4] for 10 min and then stimulated for 2 min with 50 mM KCl in the presence of 10 mg/mL horseradish peroxidase (HRP, Cat # P8250). After washout of HRP, either penetratin‐tagged peptides or dynasore (Cat # D7693) were added at concentrations described in the figure legends. Cultures were then immediately stimulated with two consecutive 30‐s applications of 50 mM KCl. Cultures were then left to rest for 30 min. Cultures were fixed in 2% glutaraldehyde in phosphate‐buffered saline at one of the three fixation time points, either directly after HRP loading (Load), after unloading (Unload) or after the 30 min rest period (Rest). After washing with 100 mM Tris (pH 7.4), cultures were exposed to 0.1% diaminobenzidine (Cat # D8001) and 0.2% H_2_O_2_ (Cat # H1009) in 100 mM Tris until colour developed. Cultures were then washed with 100 mM Tris and stained with 1% osmium tetroxide for 30 min. Samples were then dehydrated using an ethanol series and polypropylene oxide and embedded using Durcupan. Samples were sectioned, mounted on grids and viewed using an FEI Tecnai 12 transmission electron microscope (Thermo Fischer Scientific, Loughborough, UK). Nerve terminals were included in the analysis providing they contained small SVs, regardless of whether they contained HRP. Intracellular structures that were < 100 nm in diameter were arbitrarily designated to be SVs, whereas larger structures were considered endosomes. The average endosome diameter was obtained by taking the average of the longest and shortest diameters of individual endosomes using ImageJ (National Institutes of Health, Bethesda, MD, USA). In some cases, the results displayed in Figs 1, 3, 5 and 7 were part of the same experiment, therefore the same control values are presented.

### Fluorescence imaging of SV pool replenishment

**Figure 2:**
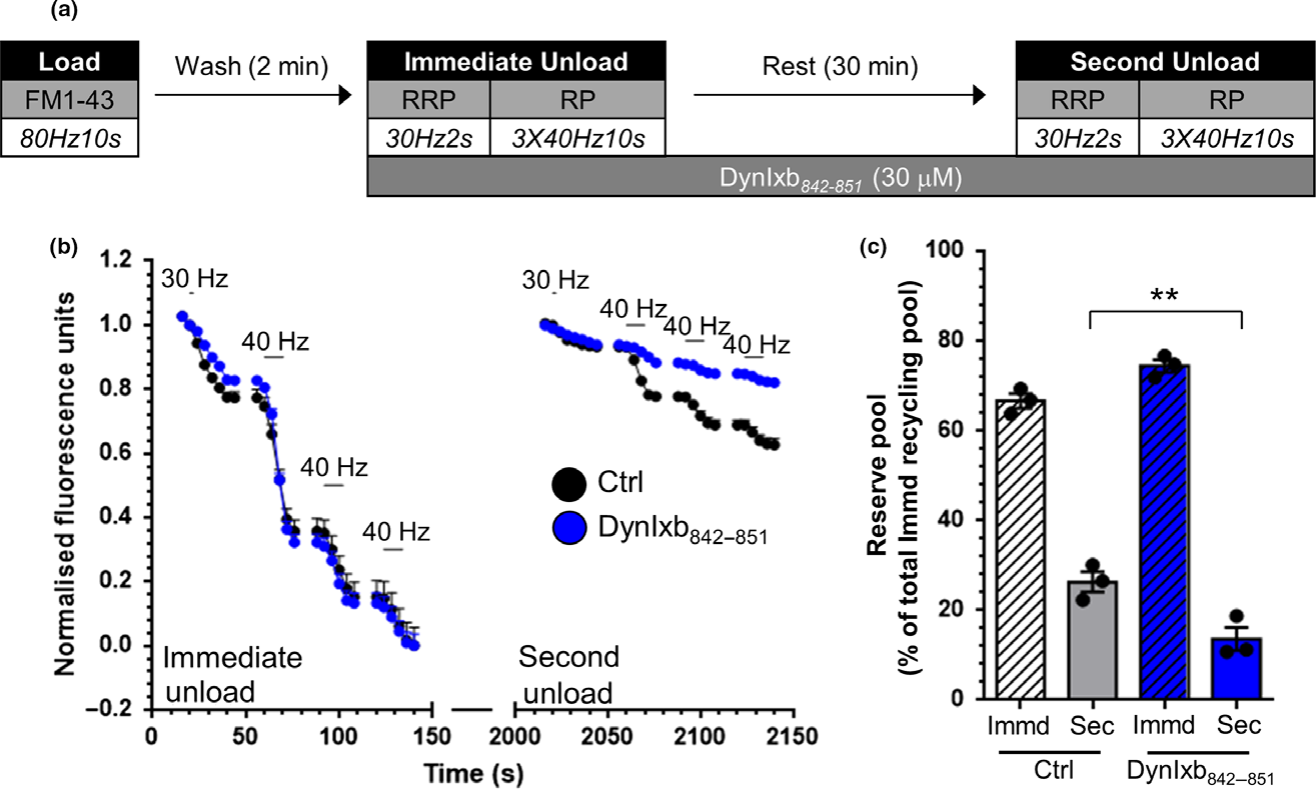
The replenishment of the reserve pool by bulk endosome‐derived synaptic vesicles requires calcineurin interactions. (a) Cultures were loaded with FM1‐43 (10 µM) using a train of 800 action potentials (80 Hz) and washed for 2 min to remove excess dye. Where indicated cultures were incubated with DynIxb_842–851_ peptide (30 µM) immediately after this wash step. The readily releasable pool (RRP) and reserve pool (RP) were sequentially unloaded using 60 action potentials (30 Hz) followed by three trains of 400 action potentials (40 Hz, Immediate Unload). The same unloading stimuli were delivered after a 30 min rest period (Second Unload). (b) Representative traces in arbitrary fluorescence units are shown for the unloading of FM1‐43 in cells without (Black) or with (Blue) DynIxb_842–851_ peptide. Bars indicate the period of stimulation. (c) Mean RP size normalized to the total immediate recycling pool is plotted for both immediate and second unloads ± SEM (all *n* = 3, from 2 culture preparations, **p < 0.01, one‐way anova).

Fluorescence imaging of SV pool replenishment was performed as previously described (Cheung and Cousin 2012). Briefly, cultures were repolarized for 10 min in incubation medium and then mounted in an imaging chamber (RC‐21BRFS; Warner Instruments, Hamden, CT, USA). Invaginating membrane was labelled with FM1‐43 (10 µM) by evoking SV turnover using electrical field stimulation delivered using platinum wires embedded in the imaging chamber (800 action potentials at 80 Hz, 100 mA, 1 ms pulse width). After washout of excess FM dye, either penetratin‐tagged peptides or dynasore were added at concentrations described in the figure legends. Cultures were then immediately stimulated (Immediate Unload) with sequential trains of action potentials to first unload the RRP (30 Hz for 2 s) and then the reserve pool (three trains of 40 Hz for 10 s). After a 30‐min rest period, an identical unloading protocol was repeated (Second Unload). This protocol allows quantification of newly generated SVs, which replenish the RRP and reserve pool. The average fluorescence drop for each unloading step was expressed as a percentage of the total SV recycling pool (RRP plus reserve pool) of the Immediate Unload, allowing comparison across multiple experiments. The start points of the second unload were then realigned to 1. Fluorescent signals were visualized using a Zeiss (Cambridge, UK) AxioObserver A1 epifluorescence microscope. FM dye loading and unloading was monitored at 500 nm excitation (emission > 535 nm) using a 20× air objective. All images were acquired using a Zeiss AxioCam CCD Camera controlled by a Zeiss AxioVision Rel. software. Time‐lapse images were acquired at 4 s intervals. The results displayed in Figs 2 and 6 were part of the same experiment, therefore the same control values are presented in both figures.

### Statistical analysis

No sample size calculation or sample outlier test was performed. A normality test was performed on the data (D’Angostino & Pearson normality test). All of the HRP datasets were normal, whereas the FM1‐43 dye datasets had too small a sample size to perform this task. Experiments from individual coverslips are indicated by *n*, with the number of independent culture preparations reported in the figure legends. All statistical analyses were performed using Microsoft Excel (Redmond, WA, USA) and GraphPad Prism 6 software (San Diego, CA, USA). Data were analysed by one‐way analysis of variance (anova) using a Tukey’s multiple comparison test for *post hoc* analysis. The one exception was the measurement of bulk endosome size, which was analysed using a two‐way anova, with a Sidak’s multiple comparison test for *post hoc* analysis. No blinding was performed in this study. All data are reported as mean ± standard error of the mean (SEM).

## Results

### Calcineurin localization at bulk endosomes is required to SV generation

The release of accumulated extracellular calcium from bulk endosomes during their acidification is essential for SV generation from these organelles (Cheung and Cousin 2013). This acidification‐dependent calcium release activates the calcium‐dependent protein phosphatase calcineurin, an event which is also essential for SV budding (Cheung and Cousin 2013). This calcium efflux is highly localized (Cheung and Cousin 2013), suggesting that calcineurin must be located in close proximity to bulk endosomes to allow its activation. Calcineurin interacts with a number of proteins via a docking motif with the consensus sequence PxIxI[TS] (where x is any amino acid; Aramburu *et al.* 1998; Aramburu *et al.* 1999; Dell'Acqua *et al.* 2002; Czirjak *et al.* 2004; Czirjak and Enyedi 2006; Filosto *et al.* 2010). Therefore, we first investigated the effect of delocalizing calcineurin from bulk endosomes using a competitive peptide that contains this motif. The sequence employed was derived from an alternatively spliced variant of dynamin I (DynIxb) which interacts with calcineurin (residues 842–851, GVPRITISDP, DynIxb_842–851_). This peptide disrupts the calcineurin–dynamin I interaction in nerve terminals (Xue *et al.* 2011). A mutant peptide was also generated as a control, which had no effect on calcineurin binding (GVARATASDP, DynIxb_842–851AAA_) (Xue *et al.* 2011). Both peptides were tagged with a penetratin entry sequence (RRMKWKK) to facilitate delivery into neuronal cultures (Cousin *et al.* 2003; Clayton *et al.* 2009; Xue *et al.* 2011).

We monitored the effect of disrupting the localization of calcineurin from bulk endosomes with the DynIxb_842–851_ peptide via a simple morphological assay (Cheung and Cousin 2012; Cheung and Cousin 2013). In this assay, both CME and ADBE were triggered using a maximal depolarizing stimulus (50 mM KCl) in the presence of the fluid‐phase marker HRP (Load, Fig. 1a). SVs and bulk endosomes that were generated by CME and ADBE, respectively, are visible as HRP‐labelled structures when examined by electron microscopy (Fig. 1b–g). To visualize generation of SVs from HRP‐labelled bulk endosomes, the existing pool of HRP‐labelled SVs was first depleted using two sequential stimuli of 50 mM KCl (Unload, Fig. 1c). After a 30‐min rest period, new HRP‐labelled SVs appeared in nerve terminals (Rest, Fig. 1d). These HRP‐labelled SVs originate from bulk endosomes, since these structures are the only remaining source of HRP within neurons. Therefore, this protocol allows tracking of HRP‐labelled SVs specifically generated from bulk endosomes during a defined time window.

Since the DynIxb_842–851_ peptide disrupts the formation of bulk endosomes during ADBE (Xue *et al.* 2011), it was applied after HRP loading was complete. This was also the case for parallel experiments using the non‐calcineurin‐binding control peptide – DynIxb_842–851AAA_ (Fig. 1a). Neither peptide had any effect on the immediate KCl‐evoked unloading of HRP‐labelled SVs, confirming the lack of role for calcineurin in SV exocytosis (Fig. 1h) (Clayton *et al.* 2009). The DynIxb_842–851AAA_ control peptide had no effect on the generation of new HRP‐labelled SVs during the 30‐min rest period when compared with control (Fig. 1h). In contrast, the DynIxb_842–851_ peptide robustly inhibited the generation of new SVs from existing HRP‐labelled bulk endosomes (Fig. 1h). Therefore, it appears that the localization of calcineurin to bulk endosomes is essential for SV generation from these organelles.

During the generation of SVs, bulk endosomes donate membrane, resulting in a reduction in their size. Therefore, to corroborate our results, we also monitored the size of HRP‐labelled bulk endosomes over the 30‐min rest period. During the SV budding process, the number of small endosomes significantly increases when compared to the ‘Load’ condition (Fig. 1i – Endosomes with diameter 100–150 nm (% of total): Load, 23.4 ± 0.9; Immediate Unload, 34.1 ± 3.2; Rest, 48.7 ± 6.5; *n* = 6 for all, *p* < 0.01 for Rest against Load, one‐way anova). The DynIxb_842–851_ peptide significantly reduced the number of small endosomes during the Rest period, confirming an inhibition of SV generation (Fig. 1j). In contrast, the control DynIxb_842–851AAA_ peptide had no significant effect on the appearance of small bulk endosomes over the 30‐min rest period (Fig. 1j). Therefore, the inhibition of both SV generation and reduction in bulk endosome diameter suggests that localization of calcineurin at bulk endosomes is required for SV budding to proceed.

Bulk endosome‐derived SVs specifically replenish the reserve pool of SVs after high‐intensity stimulation (Richards *et al.* 2003; Cheung *et al.* 2010). Therefore, we next determined whether the disruption of calcineurin localization from bulk endosomes perturbed the replenishment of the reserve pool of SVs. To assay reserve pool refilling by bulk endosome‐derived SVs, both CME and ADBE were triggered with a train of 800 action potentials (80 Hz) in the presence of the dye FM1‐43 (Fig. 2a). FM1‐43‐loaded SVs were then immediately depleted after dye loading by sequentially unloading the RRP (60 action potentials at 30 Hz) and then the reserve pool (three 400 action potential trains at 40 Hz). Cultures were then rested for 30 min to allow SV generation from bulk endosomes, and their subsequent replenishment of SV pools. The RRP and reserve pool were then mobilized again, with the majority of released fluorescence originating from the reserve pool (Fig. 2b).

In a similar manner to the ultrastructural assay, DynIxb_842–851_ was applied after the FM1‐43 load, but before the immediate unloading of FM1‐43‐labelled SVs (Fig. 2a). Incubation with the DynIxb_842–851_ peptide had no effect on the immediate unloading of SVs, but it severely disrupted replenishment of the reserve pool during the 30‐min rest period (Fig. 2b and c). Thus, the localization of calcineurin at bulk endosomes is essential for both the generation of SVs and their subsequent replenishment of the reserve pool.

### Dynamin I GTPase activity is required for SV generation from bulk endosomes

**Figure 3:**
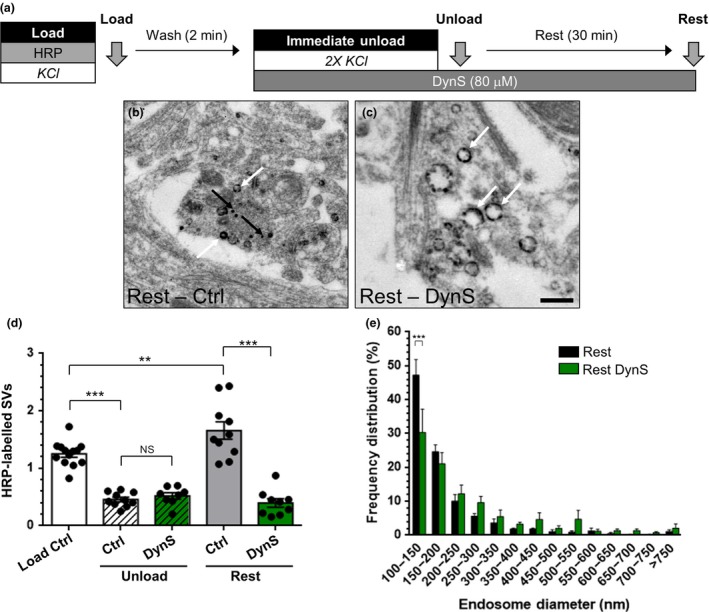
Dynamin I GTPase activity is required for synaptic vesicle (SV) budding from bulk endosomes. (a) Cultures were loaded with horse radish peroxidase (HRP) (10 mg/mL) for 2 min in the presence of KCl (50 mM) and washed immediately to remove excess HRP. Where indicated cultures were incubated with dynasore (DynS, 80 µM) immediately after this wash step. Neurons were then stimulated twice with KCl (50 mM, 30 s each) to release all available SVs (Immediate Unload) and left to rest for 30 min. Cultures were either fixed after HRP loading (Load), the immediate unload (Unload) or the rest period (Rest) as indicated by arrows. (b, c) Representative electron micrographs of the treatments described above are shown (b – Rest, c – Rest DynS; scale bar – 500 nm). Black and white arrows indicate HRP‐labelled SVs and endosomes respectively. (d) Bar graph displays the mean number of HRP‐labelled SVs per nerve terminal ± SEM in either Load, Immediate Unload (hatched bars) and Rest (solid bars) conditions in the presence or absence of DynS. Number of experiments: Load Ctrl *n* = 13, Unload Ctrl *n* = 10, Unload DynS *n* = 8, Rest Ctrl *n* = 10, Rest DynS *n* = 9, from three culture preparations **p < 0.01, ***p < 0.001 one‐way anova). (e) Frequency distribution of endosome diameter for Rest in the presence or absence of DynS. The number of HRP‐labelled endosomes were as follows: Rest Ctrl *n* = 986; Rest DynS *n* = 759; Rest Ctrl *n* = 10, Rest DynS *n* = 9, from 3 culture preparations (***p < 0.001 two‐way anova).

The inhibitory DynIxb_842–851_ peptide is derived from an alternatively spliced form of dynamin I (DynIxb) (Xue *et al.* 2011). Therefore, we next tested whether dynamin I itself could be required for SV generation at bulk endosomes. We first determined whether its GTPase activity was required for SV budding. To achieve this, we examined the effect of the dynamin antagonist dynasore in our ultrastructural HRP assay (Macia *et al.* 2006; Newton *et al.* 2006). Dynasore (80 µM) was added after KCl‐evoked HRP loading to ensure the SV budding process was decoupled from essential dynamin‐dependent fission events at the plasma membrane (Newton *et al.* 2006; Ferguson *et al.* 2007; Clayton *et al.* 2009; Kononenko *et al.* 2014) (Fig. 3a–c). Dynasore had no effect on the KCl‐evoked fusion of HRP‐labelled SVs during the immediate unload, confirming no role in SV exocytosis (Fig. 3d). In contrast, the drug abolished the production of HRP‐labelled SVs from previously generated bulk endosomes (Fig. 3d), suggesting dynamin I GTPase activity was essential for SV budding. In agreement, when the size of HRP‐labelled bulk endosomes were assessed after 30 min in the presence of dynasore, a dramatic decrease in the number of small endosomes was observed (Fig. 3e). Thus using two independent measurements, dynamin I GTPase activity appears to be essential for SV generation from bulk endosomes.

**Figure 4:**
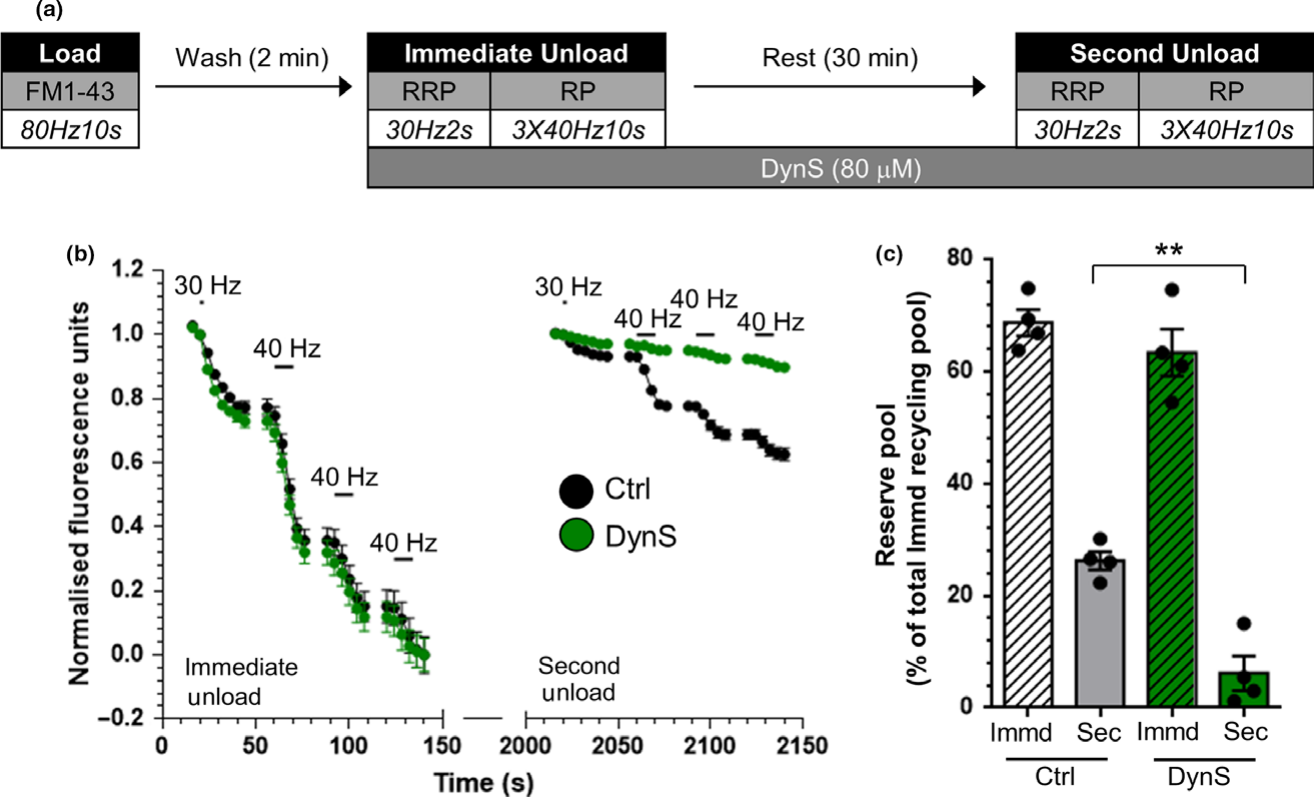
Dynamin I GTPase activity is required for replenishment of the reserve pool by bulk endosome‐derived synaptic vesicles. (a) Cultures were loaded with FM1‐43 (10 µM) using a train of 800 action potentials (80 Hz) and washed for 2 min to remove excess dye. Where indicated cultures were incubated with dynasore (DynS, 80 µM) immediately after this wash step. The readily releasable pool (RRP) and reserve pool (RP) were sequentially unloaded using 60 action potentials (30 Hz) followed by three trains of 400 action potentials (40 Hz, Immediate Unload). The same unloading stimuli were delivered after a 30 min rest period (Second Unload). (b) Representative traces in arbitrary fluorescence units are shown for the unloading of FM1‐43 in cells without (Black) or with (Green) DynS. Bars indicate the period of stimulation. (c) Mean RP size normalized to the total immediate recycling pool is plotted for both immediate and second unloads ± SEM (Ctrl *n* = 4, DynS *n* = 4, from 2 culture preparations **p < 0.01, one‐way anova).

We next determined whether inhibition of dynamin I GTPase activity also perturbed the replenishment of the reserve pool of SVs. As with the HRP assay, dynasore was applied after dye loading was complete to ensure no interference with plasma membrane endocytic events (Fig. 4a). The drug had no effect on the fusion of CME‐derived SVs during the immediate unload stimulus, confirming a lack of effect on SV exocytosis (Fig. 4b). In contrast the replenishment of the reserve pool during the 30‐min rest period was almost completely ablated (Fig. 4c). Thus inhibition of dynamin I GTPase activity abolishes both the generation of new SVs from bulk endosomes and their subsequent replenishment of the reserve pool. This indicates that SV fission from bulk endosomes is most likely to be mediated by dynamin I GTPase activity.

### The phosphorylation‐dependent dynamin I–syndapin I interaction is required for SV generation from bulk endosomes

Calcineurin dephosphorylates dynamin I during high‐intensity stimulation in neurons, resulting in an increased association with the endocytosis protein syndapin I (Anggono *et al.* 2006; Clayton *et al.* 2009). The major phosphorylation sites on dynamin I (Ser‐774 and Ser‐778) encompass part of the syndapin I interaction site, explaining its phosphoregulation (Anggono *et al.* 2006; Anggono and Robinson 2007). This means that the dynamin I–syndapin I interaction can be disrupted by competitive peptides which mimic the dephosphoryated site (Ser774/778Ala; DynI_769–784_AA), whereas peptides which mimic the phosphorylated site (Ser774/778Glu; DynI_769–784_EE) have no effect (Anggono *et al.* 2006). Since dynamin I may localize calcineurin at bulk endosomes and is a major calcineurin substrate, we next investigated whether the dynamin I–syndapin I interaction is required for SV generation.

**Figure 5:**
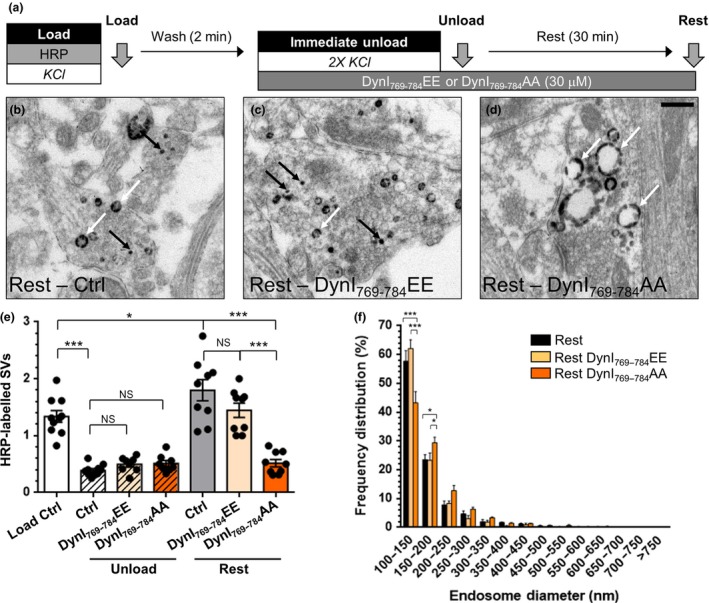
Disruption of the dynamin I–syndapin I interaction arrests synaptic vesicle (SV) budding from bulk endosomes. (a) Cultures were loaded with horse radish peroxidase (HRP) (10 mg/mL) for 2 min in the presence of KCl (50 mM) and washed immediately to remove excess HRP. Where indicated cultures were incubated with either DynI_769–784_AA or DynI_769–784_EE peptides (both 30 µM) immediately after this wash step. Neurons were then stimulated twice with KCl (50 mM, 30 s each) to release all available SVs (Immediate Unload) and left to rest for 30 min. Cultures were either fixed after HRP loading (Load), the immediate unload (Unload) or the rest period (Rest) as indicated by arrows. (b–d) Representative electron micrographs of the treatments described above are shown (b – Rest, c – Rest DynI_769–784_EE, d – Rest DynI_769–784_AA; scale bar – 500 nm). Black and white arrows indicate HRP‐labelled SVs and endosomes respectively. (e) Bar graph displays the mean number of HRP‐labelled SVs per nerve terminal ± SEM in either Load, Immediate Unload (hatched bars) and Rest (solid bars) conditions in the presence or absence of peptides. Number of experiments: *n* = 10 for Load Ctrl and Unload Ctrl, *n* = 9 for Rest Ctrl, Rest DynI_769–784_EE and Rest DynI_769–784_AA, *n* = 8 for Unload DynI_769–784_EE and Rest DynI_769–784_AA from three culture preparations *p < 0.05, ***p < 0.001 one‐way anova). (f) Frequency distribution of endosome diameter for Rest with competitive peptides. The number of HRP‐labelled endosomes were as follows: Rest Ctrl *n* = 1585; Rest DynI_769–784_EE *n* = 801; Rest DynI_769–784_AA *n* = 1571, *n* = 9 coverslips for Rest Ctrl, Rest DynI_769–784_EE and Rest DynI_769–784_AA from three culture preparations (*p < 0.05, ***p < 0.001, two‐way anova).

To determine the effect of DynI_769–784_AA and DynI_769–784_EE peptides on generation of SVs from bulk endosomes, we performed our HRP budding assay. Peptides were added after the HRP load, to exclude potential effects on ADBE (Fig. 5a). Neither DynI_769–784_AA nor DynI_769–784_EE had any effect on the fusion of HRP‐labelled SVs during the immediate unloading stimulus (Fig. 5b–e). DynI_769–784_EE also had no effect on the generation of new HRP‐labelled SVs from bulk endosomes during the 30‐min rest period (Fig. 5e). In contrast, DynI_769–784_AA significantly inhibited the production of HRP‐labelled SVs from bulk endosomes (Fig. 5e). Furthermore, DynI_769–784_AA significantly reduced the number of small HRP‐labelled endosomes that were present after 30 min, whereas DynI_769–784_EE had no effect (Fig. 5f). Therefore, inhibition of the phosphorylation‐dependent dynamin I–syndapin I interaction perturbs SV generation from bulk endosomes.

**Figure 6:**
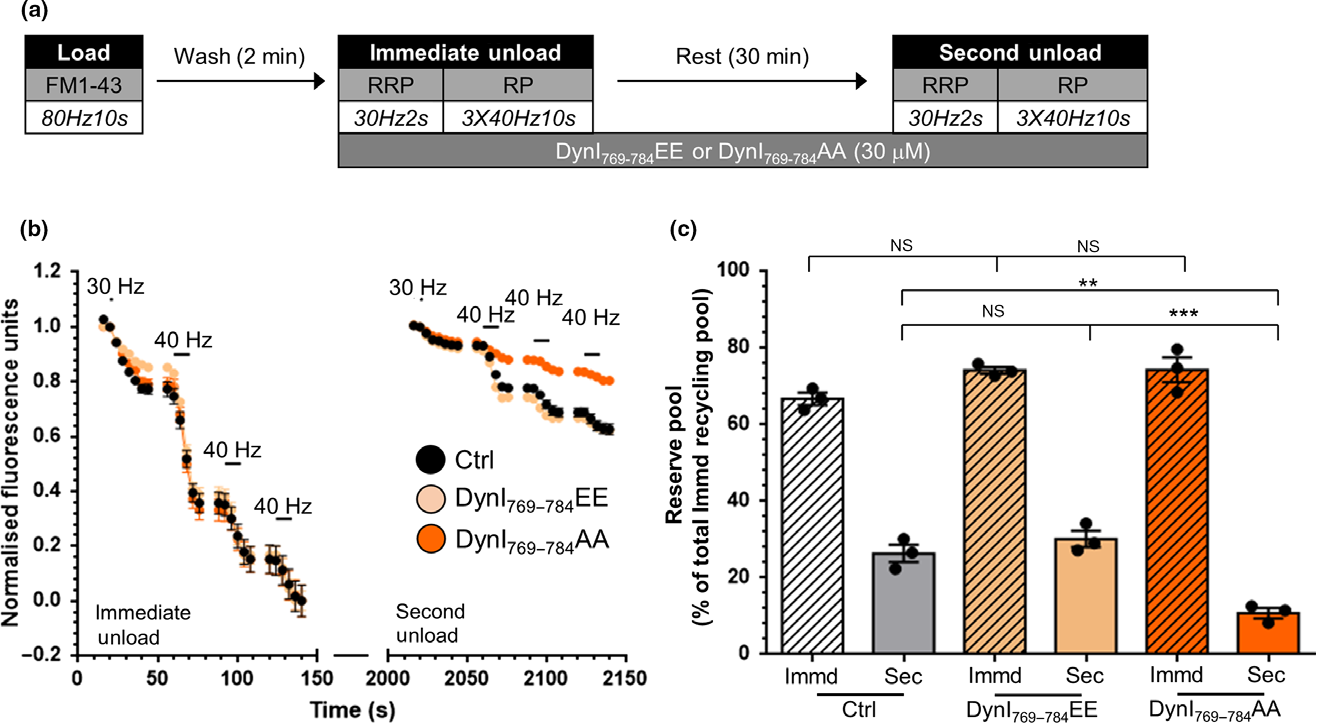
The replenishment of the reserve pool by bulk endosome‐derived synaptic vesicles requires the dynamin I–syndapin I interaction. (a) Cultures were loaded with FM1‐43 (10 µM) using a train of 800 action potentials (80 Hz) and washed for 2 min to remove excess dye. Where indicated cultures were incubated with either DynI_769–784_AA or DynI_769–784_EE peptides (30 µM) immediately after this wash step. The readily releasable pool (RRP) and reserve pool (RP) were sequentially unloaded using 60 action potentials (30 Hz) followed by three trains of 400 action potentials (40 Hz, Immediate Unload). The same unloading stimuli were delivered after a 30 min rest period (Second Unload). (b) Representative traces in arbitrary fluorescence units are shown for the unloading of FM1‐43 in cells without peptides (Black), DynI_769–784_EE (light orange) or DynI_769–784_AA (Orange) peptides. Bars indicate the period of stimulation. (c) Mean RP size normalized to the total immediate recycling pool is plotted for both immediate and second unloads ± SEM (all *n* = 3; from 2 culture preparations **p < 0.01, ***p < 0.001, one‐way anova).

We then determined whether the dynamin I–syndapin I interaction is also required for replenishment of the reserve SV pool (Fig. 6a). Either DynI_769–784_AA or DynI_769–784_EE were applied to cultures after loading of FM1‐43. Neither peptide affected the fusion of FM1‐43‐loaded SVs during the immediate unloading stimulus (Fig. 6b). DynI_769–784_EE had no effect on the replenishment of the reserve pool, confirming its lack of effect on SV generation from bulk endosomes (Fig. 6c). In contrast, DynI_769–784_AA produced a significant inhibition of reserve pool replenishment over the 30‐min rest period (Fig. 6c). Thus, the calcineurin‐mediated, dephosphorylation‐dependent interaction between dynamin I and syndapin I is required for SV generation from bulk endosomes.

### Syndapin I–EH domain interactions are not required for SV generation from bulk endosomes

**Figure 7:**
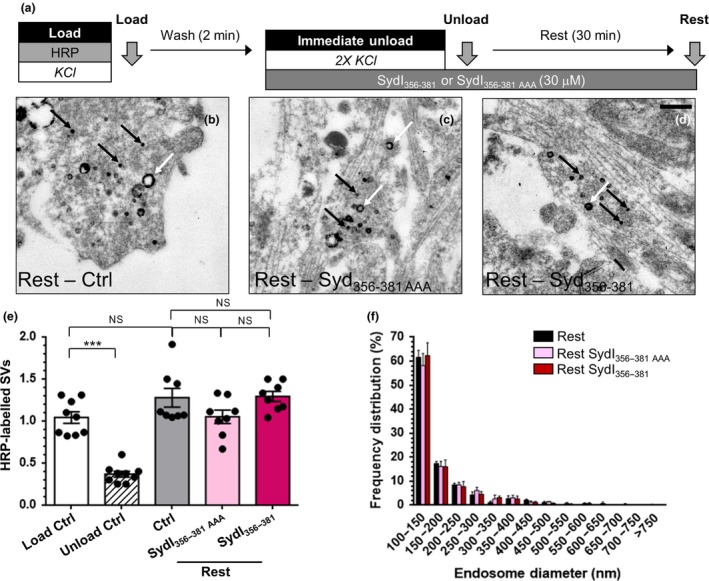
Syndapin I NPF interactions are not required for synaptic vesicle (SV) budding from bulk endosomes. (a) Cultures were loaded with horse radish peroxidase (HRP) (10 mg/mL) for 2 min in the presence of KCl (50 mM) and washed immediately to remove excess HRP. Where indicated cultures were incubated with either SydI_356–381_ or SydI_356–381AAA_ peptides (both 30 µM) immediately after this wash step. Neurons were then stimulated twice with KCl (50 mM, 30 s each) to release all available SVs (Immediate Unload) and left to rest for 30 min. Cultures were either fixed after HRP loading (Load), the immediate unload (Unload) or the rest period (Rest) as indicated by arrows. (b–d) Representative electron micrographs of the treatments described above are shown (b – Rest, c – Rest SydI_356–381AAA_, d – Rest SydI_356–381_; scale bar – 500 nm). Black and white arrows indicate HRP‐labelled SVs and endosomes respectively. (e) Bar graph displays the mean number of HRP‐labelled SVs per nerve terminal ± SEM in either Load, Immediate Unload (hatched bars) and Rest (solid bars) conditions in the presence or absence of peptides. Number of experiments: all *n* = 9 Load Ctrl and Unload Ctrl, *n* = 8 Rest Ctrl, Rest SydI_356–381AAA_ and Rest SydI_356–381_. All from three culture preparations; ***p < 0.001 one‐way anova). (f) Frequency distribution of endosome diameter for Rest with competitive peptides. The number of HRP‐labelled endosomes were as follows: Rest Ctrl *n* = 605; Rest SydI_356–381AAA_ *n* = 761; Rest SydI_356–381_ *n* = 478, *n* = 8 coverslips for Rest Ctrl, Rest SydI_356–381AAA_ and Rest SydI_356–381_ from three culture preparations (all non‐significant (ns), two‐way anova).

One key function of the ubiquitous isoform of syndapin, syndapin II, is the control of vesicle budding from early endosomes in non‐neuronal cells (Braun *et al.* 2005). A key interaction in this control is with EHDs, which are ATPases required for vesicle fission from intracellular organelles in a number of cell systems (Daumke *et al.* 2007; Naslavsky and Caplan 2011) and are expressed in central nerve terminals (Braun *et al.* 2005; Wei *et al.* 2010). Syndapin I interacts with EHD proteins via two NPF repeats located between its F‐BAR and SH3 domains (Braun *et al.* 2005). Therefore, to determine whether the syndapin I‐dependent recruitment of EHD proteins was essential for SV generation from bulk endosomes, this interaction was inhibited by a competitive peptide mimicking the EHD interaction site (SydI_356–381_). A control peptide where the two NPF repeats were substituted for alanine was used as a control (SydI_356–381AAA_). The effect of these peptides in the HRP‐budding assay was then examined. The experiment was performed as described previously, with the inhibitory peptides added after the initial KCl loading stimulus (Fig. 7a). Neither peptide had an effect on the KCl‐evoked immediate unloading of HRP SVs, confirming a lack of effect on SV exocytosis (Fig. 7b–e). The control SydI_356–381AAA_ peptide also had no significant effect on SV generation from HRP‐labelled bulk endosomes (Fig. 7e). This was also the case for the SydI_356–381_ peptide (Fig. 7e), suggesting no role for EHD interactions in SV generation from bulk endosomes. The lack of effect of either peptide was confirmed when the diameter of HRP‐labelled bulk endosomes was assayed after the 30‐min rest period (Fig. 7f). Therefore, syndapin‐dependent interactions with EHD proteins have no role in SV generation from activity‐dependent bulk endosomes.

## Discussion

The generation of SVs from bulk endosomes after intense periods of activity is essential to sustain neurotransmission (Nicholson‐Fish *et al.* 2015). This process relies on the efflux of accumulated calcium from bulk endosomes during their acidification (Cheung and Cousin 2013). We have shown here that a calcineurin‐dependent dephosphorylation event (the evoked interaction between dynamin I and syndapin I) is essential for SV generation and reveals multiple roles for this interaction in the ADBE pathway.

We observed SV production directly using a well‐characterized morphological assay that specifically tracks SVs generated by ADBE (Cheung and Cousin 2012; Cheung and Cousin 2013). In this assay, elevated KCl was used to both evoke ADBE and then to deplete HRP‐loaded SVs. We acknowledge that action potential stimulation (which permits membrane repolarization between pulses) is more physiologically relevant to the clamped depolarization evoked by KCl. However, for technical reasons we employed KCl depolarization in this instance. In support of its use, application of elevated KCl is equivalent to the delivery of 800 action potentials (80 Hz) in evoking a number of pre‐synaptic processes including; extent of SV exocytosis (Cousin and Evans 2011); the extent of both ADBE and CME (Clayton *et al.* 2009) and the replenishment of both the RRP and reserve pools (Cheung and Cousin 2012; Cheung and Cousin 2013). We also employed a reserve pool replenishment assay to corroborate our results, since ADBE‐derived SVs selectively refill this pool (Richards *et al.* 2003; Cheung *et al.* 2010; Cheung and Cousin 2012; Cheung and Cousin 2013). In all cases where generation of SVs from bulk endosomes was inhibited, we observed a parallel impact on reserve SV pool replenishment.

In this study, we exploited a series of peptides that disrupt specific interactions within central nerve terminals (Anggono *et al.* 2006; Xue *et al.* 2011). All peptides were tagged with the penetratin sequence, which is derived from the third α‐helix of the *Drosophila* Antennopedia homeodomain protein (that facilitates peptide access into cells and neurons; Lindgren *et al.* 2000; Cousin *et al.* 2003). The advantage of this acute form of intervention is that it decouples the role of these interactions in endocytosis from SV generation from endosomes in a manner that is not possible with either knockdown or over‐expression vectors. This is particularly important since calcineurin, dynamin I and syndapin I all play key roles in bulk endosome generation (Evans and Cousin 2007; Clayton *et al.* 2009; Xue *et al.* 2011; Wu *et al.* 2014a).

One other acute intervention is the inhibition of dynamin I GTPase activity via the antagonist dynasore (Macia *et al.* 2006). The widespread use of this drug and derivatives such as dyngo‐4a (McCluskey *et al.* 2013), has indicated that almost all forms of SV endocytosis at the pre‐synaptic plasma membrane are dynamin‐mediated (Newton *et al.* 2006; Clayton *et al.* 2009; Watanabe *et al.* 2013; McCluskey *et al.* 2013). However, the demonstration that these compounds inhibit endocytosis in cells lacking all three dynamin isoforms (Park *et al.* 2013), suggest that any observed effects must be treated with caution. Nevertheless, the presence of dynamin I on purified bulk endosomes (Kokotos *et al.* 2018), combined with the inhibitory effect of dynamin I‐derived peptides, suggests that this GTPase performs an important role in SV generation from these compartments.

The obligatory requirement for dynamin I in bulk endosome fission remains a matter of debate. We have shown via the combined use of dominant‐negative dynamin I mutants, competitive peptides and pharmacological inhibition that dynamin I plays an important role in this process (Clayton *et al.* 2009). In support, siRNA‐mediated knockdown of dynamin I and III only perturbed endocytosis during high‐frequency stimulation, suggesting a key role in ADBE (Kononenko *et al.* 2014). In addition, a role for dynamin I in bulk endosome fission was demonstrated using either acute photoinactivation at *Drosophila* neuromuscular junctions (Kasprowicz *et al.* 2014) or temperature‐sensitive mutations in *C. elegans* (Kittelmann *et al.* 2013). In both studies, endosomes were formed but could not detach from the plasma membrane. In contrast, a form of fluid‐phase retrieval very similar to ADBE occurs in dynamin I/III double knockout nerve terminals (Hayashi *et al.* 2008; Wu *et al.* 2014b). Importantly, the generation of SVs from these endosomes is severely disrupted in either dynamin I or dynamin I/III double knockout neurons (Wu *et al.* 2014b). Furthermore, there is an almost complete absence of SV budding from bulk endosomes in temperature‐sensitive *C. elegans* dynamin I mutants (Kittelmann *et al.* 2013). Therefore, it appears that dynamin I is required for SV generation from bulk endosomes, even if its role in bulk endosome formation is still debated.

Calcineurin is located on bulk endosomes (Kokotos *et al.* 2018) and plays a key role in SV generation from these organelles (Cheung and Cousin 2013). We showed that SV generation was disrupted when a competitive peptide that interferes with calcineurin interactions was applied to neurons, indicating that calcineurin must be located closely to sites of calcium efflux on bulk endosomes. This peptide was derived from a specific splice variant of dynamin (Dynamin Ixb; Xue *et al.* 2011), therefore it is possible that dynamin I is the protein that localizes calcineurin to endosomes. However, since the peptide employed would disrupt any calcineurin interaction mediated by the PRITIS motif (Aramburu *et al.* 1998; Aramburu *et al.* 1999; Dell'Acqua *et al.* 2002; Czirjak *et al.* 2004; Czirjak and Enyedi 2006; Filosto *et al.* 2010) we cannot conclusively state this from these experiments alone.

The activity‐dependent activation of calcineurin and subsequent dephosphorylation of dynamin I and interaction with syndapin I is also essential for bulk endosome generation (Clayton *et al.* 2009). Interestingly in this series of experiments, we observe much more dramatic effects on SV generation than with the same or very similar interventions at the plasma membrane, suggesting that SV generation may be the most critical role for calcineurin‐mediated events in ADBE. In agreement, assays that monitor production of release‐ready SVs after both bulk endosome budding and SV generation, closely track the inhibitory effects of blocking SV generation alone (Kumashiro *et al.* 2005; Evans and Cousin 2007; Clayton *et al.* 2009). Therefore, the principal role of this calcium‐dependent dephosphorylation cascade may be at the bulk endosome, rather than at the plasma membrane.

The downstream mechanism via which this cascade triggers SV generation remains to be determined. The presence of dynamin I on bulk endosomes (Kokotos *et al.* 2018) and the potential requirement for its GTPase activity, suggests that it may be the key fission mediator. However, this does not explain the requirement for its interaction with syndapin I in the process. Syndapin I is located on bulk endosomes (Kokotos *et al.* 2018) and its chelation by intracellular antibodies disrupts SV generation from bulk endosomes in Lamprey nerve terminals (Andersson *et al.* 2008). Syndapin I is modular protein with a lipid‐deforming F‐BAR domain, central NPF repeats and a C‐terminal SH3 domain (Qualmann *et al.* 1999). We chose to disrupt syndapin I interactions acutely using competitive peptides, since expressing syndapin mutants would also disrupt bulk endosome formation (Clayton *et al.* 2009; Cheung *et al.* 2010). The central NPF repeats recruit EHD proteins to mediate vesicle fission in a number of endosomal systems (Braun *et al.* 2005; Naslavsky and Caplan 2011). Furthermore, EHD proteins are suggested to control dynamin helix assembly and thus SV fission (Jakobsson *et al.* 2011). However, when interactions between syndapin I and EHD proteins were perturbed with competitive peptides encompassing the NPF repeats, there was no effect on SV generation. This suggests syndapin I–EHD interactions do not play a role in SV generation from bulk endosomes. The F‐BAR domain of syndapin I induces the deformation of lipids that may aid SV generation (Wang *et al.* 2009). Furthermore, the syndapin I F‐BAR domain exhibits a binding preference for shallow curved membranes (such as on endosomes) rather than the more tight curvature of SVs (Shimada *et al.* 2007; Henne *et al.* 2007). The lipid‐deforming activity of syndapin I is auto‐inhibited via an internal interaction with its SH3 domain, and importantly binding of dynamin I to this SH3 domain releases this inhibition to trigger lipid deformation (Rao *et al.* 2010; Goh *et al.* 2012). Therefore, we propose a model where the calcineurin‐mediated dephosphorylation of dynamin I triggers an interaction with the syndapin I SH3 domain resulting in lipid deformation, to facilitate SV generation. This is still a working model and the exact location or sequence of events in this cascade has still to be confirmed. Nevertheless, this is an attractive hypothesis for future studies, since it provides an explanation for the essential requirement for all three molecules.
